# 2-{(*E*)-4-[4-(Trifluoro­meth­yl)phen­oxy]but-2-en­yloxy}phenyl *N*-methyl­carbamate

**DOI:** 10.1107/S1600536813001347

**Published:** 2013-02-02

**Authors:** Hong-Ju Ma, Meng-Han Xu, Jian-Hua Zhang, Jian-Hong Li, Jun Ning

**Affiliations:** a College of Plant Science & Technology of Huazhong Agricultural University, Wuhan 430070, People’s Republic of China; b Key Laboratory of Pesticide Chemistry and Application, Ministry of Agriculture, Institute of Plant Protection, Chinese Academy of Agricultural Sciences, Beijing 100193, People’s Republic of China

## Abstract

In the title compound, C_19_H_18_F_3_NO_4_, which was designed and synthesized as a dual-site inhibitor of insect AChE (acetyl­cholinesterase), the dihedral angle between the methyl­carbamate group and the benzene ring is 72.47 (6)°. In the crystal, inversion dimers are linked by pairs of N—H⋯O hydrogen bonds.

## Related literature

For background to multivalent ligand-receptor inter­actions and their pharmaceutical applications, see: Carlier *et al.* (1999 ▶); Hu *et al.* (2002 ▶); Kitov *et al.* (2000 ▶); Kopytek *et al.* (2000 ▶); Kryger *et al.* (1999 ▶); Lee & Lee (1995 ▶); Luedtke *et al.* (2003 ▶); Mammen *et al.* (1998 ▶); Pang *et al.* (1996 ▶). For agrochemical applications of the cluster effect, see: Ma *et al.* (2010 ▶); Zhao *et al.* (2008 ▶, 2009 ▶). For the structure of AChE from *Torpedo californica* (TcAChe), see: Sussman *et al.* (1991 ▶); Harel *et al.* (1993 ▶).
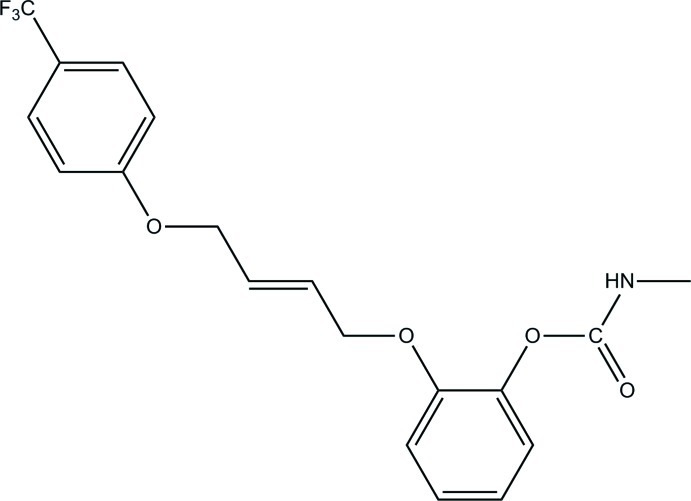


## Experimental

### 

#### Crystal data

C_19_H_18_F_3_NO_4_
*M* *_r_* = 381.34
Monoclinic, 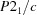

*a* = 12.413 (3) Å
*b* = 9.3936 (19) Å
*c* = 16.202 (3) Å
β = 111.65 (3)°
*V* = 1755.9 (6) Å^3^
*Z* = 4
Cu *K*α radiation
μ = 1.05 mm^−1^
*T* = 173 K
0.47 × 0.30 × 0.25 mm

#### Data collection

Rigaku R-AXIS RAPID IP area-detector diffractometer
Absorption correction: multi-scan (*ABSCOR*; Higashi, 1995 ▶) *T*_min_ = 0.639, *T*_max_ = 0.780
11613 measured reflections
3163 independent reflections
2615 reflections with *I* > 2σ(*I*)
*R*_int_ = 0.033

#### Refinement

*R*[*F* ^2^ > 2σ(*F* ^2^)] = 0.044
*wR*(*F* ^2^) = 0.107
*S* = 1.07
3163 reflections
274 parameters
60 restraints
H-atom parameters constrained
Δρ_max_ = 0.29 e Å^−3^
Δρ_min_ = −0.22 e Å^−3^

### 

Data collection: *RAPID-AUTO* (Rigaku 2001 ▶); cell refinement: *RAPID-AUTO*; data reduction: *RAPID-AUTO*; program(s) used to solve structure: *SHELXS97* (Sheldrick, 2008 ▶); program(s) used to refine structure: *SHELXL97* (Sheldrick, 2008 ▶); molecular graphics: *XP* in *SHELXTL* (Sheldrick, 2008 ▶); software used to prepare material for publication: *SHELXL97*.

## Supplementary Material

Crystal structure: contains datablock(s) I, global. DOI: 10.1107/S1600536813001347/mw2102sup1.cif

Structure factors: contains datablock(s) I. DOI: 10.1107/S1600536813001347/mw2102Isup2.hkl

Supplementary material file. DOI: 10.1107/S1600536813001347/mw2102Isup3.cml

Additional supplementary materials:  crystallographic information; 3D view; checkCIF report

## Figures and Tables

**Table 1:** Hydrogen-bond geometry (Å, °)

*D*—H⋯*A*	*D*—H	H⋯*A*	*D*⋯*A*	*D*—H⋯*A*
N1—H1⋯O2^i^	0.88	2.22	3.072 (2)	163

Symmetry code: (i) 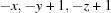.

**Fig. 1.**
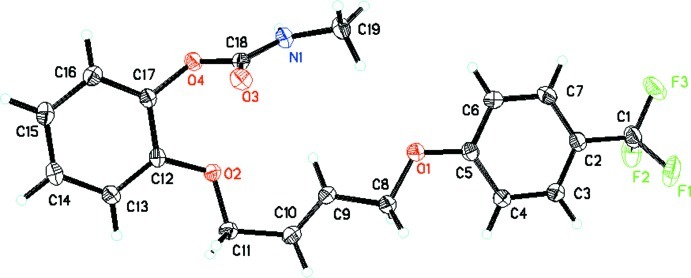
The molecular structure of the title compound showing 50% probability displacement ellipsoids and the atom-numbering scheme.

## supplementary crystallographic information

### Comment

Multivalent ligand-receptor interactions, known as the cluster effect, are defined as specific simultaneous associations of multiple ligands present on a molecular construct that bind to multiple receptors presented on a biological entity (Mammen *et al.*, 1998; Lee *et al.*, 1995). In biological systems, multivalent ligands often possess increased functional affinity for their targets compared with that of monovalent ligands (Kitov *et al.*, 2000; Kopytek *et al.*, 2000; Luedtke *et al.*, 2003). Acetylcholinesterase (AChE; EC 3.1.1.7) is a key enzyme in the nervous system, terminating nerve impulses by catalyzing the hydrolysis of the neurotransmitter acetylcholine. X-ray crystallographic structural analysis of the AChE from *Torpedo californica* (TcAChe) has demonstrated that the active site lies near the bottom of a deep, narrow gorge that reaches half way into the protein and that 14 aromatic residues line a substantial portion of the surface of the gorge (Sussman *et al.*, 1991). This cavity was named the 'active site gorge' with peripheral sites existing at the gorge mouth and Trp279 being the main site (Harel *et al.*, 1993). On the basis of the structure of AChE, many potential dual-site binding inhibitors of AChE have been synthesized (Pang *et al.*, 1996; Carlier *et al.*, 1999; Hu *et al.*, 2002) and demonstrated in drugs in order to treat or alleviate Alzheimer's disease (Kryger *et al.*, 1999). In the area of pesticide, dual- or multiple-site inhibitors of insect AChE were designed and synthesized in our research group (Zhao *et al.*., 2008; Zhao *et al*., 2009). Recently, we synthesized novel carbamate derivatives as potential dual-binding site acetylcholinesterase inhibitors (Ma *et al.*, 2010). The crystal structure of the title compound (I) is shown in Fig. 1.

### Experimental

2-((*E*)-4-(4-(trifluoromethyl)phenoxy)but-2-enyloxy)phenyl methylcarbamate (0.2 g) was dissolved in 95% ethanol (50 ml) at room temperature. Colorless crystals of compound (I) were obtained through slow evaporation after two weeks.

### Refinement

The trifluoromethyl group showed orientational disorder with two resolved alternative sites which were refined independently leading to a 71:29 occupancy ratio. All non-hydrogen atoms were refined with anisotropic displacement parameters. The carbon-bound H atoms were placed at calculated positions, with C—H = 0.93 - 0.98 Å, and were included in the refinement in the riding model approximation with *U*iso(H) set to 1.2 - 1.5*U*eq(C).

### Crystal data

**Table:**

C_19_H_18_F_3_NO_4_	*F*(000) = 792
*M**_r_* = 381.34	*D*_x_ = 1.443 Mg m^−^^3^
Monoclinic, *P*2_1_/*c*	Cu *K*α radiation, λ = 1.54178 Å
Hall symbol: -P 2ybc	Cell parameters from 468 reflections
*a* = 12.413 (3) Å	θ = 2.2–68.3°
*b* = 9.3936 (19) Å	µ = 1.05 mm^−^^1^
*c* = 16.202 (3) Å	*T* = 173 K
β = 111.65 (3)°	Block, colorless
*V* = 1755.9 (6) Å^3^	0.47 × 0.30 × 0.25 mm
*Z* = 4	

### Data collection

**Table:**

Rigaku R-AXIS RAPID IP area-detector diffractometer	3163 independent reflections
Radiation source: rotating anode	2615 reflections with *I* > 2σ(*I*)
Graphite monochromator	*R*_int_ = 0.033
ω scans at fixed χ = 45°	θ_max_ = 68.2°, θ_min_ = 3.8°
Absorption correction: multi-scan (*ABSCOR*; Higashi, 1995)	*h* = −14→14
*T*_min_ = 0.639, *T*_max_ = 0.780	*k* = −11→10
11613 measured reflections	*l* = −18→19

### Refinement

**Table:**

Refinement on *F*^2^	Secondary atom site location: difference Fourier map
Least-squares matrix: full	Hydrogen site location: inferred from neighbouring sites
*R*[*F*^2^ > 2σ(*F*^2^)] = 0.044	H-atom parameters constrained
*wR*(*F*^2^) = 0.107	*w* = 1/[σ^2^(*F*_o_^2^) + (0.0381*P*)^2^ + 0.8611*P*] where *P* = (*F*_o_^2^ + 2*F*_c_^2^)/3
*S* = 1.07	(Δ/σ)_max_ = 0.001
3163 reflections	Δρ_max_ = 0.29 e Å^−^^3^
274 parameters	Δρ_min_ = −0.22 e Å^−^^3^
60 restraints	Extinction correction: *SHELXL97* (Sheldrick, 2008), Fc^*^=kFc[1+0.001xFc^2^λ^3^/sin(2θ)]^-1/4^
Primary atom site location: structure-invariant direct methods	Extinction coefficient: 0.0033 (3)

### Special details

**Table:**

Geometry. All e.s.d.'s (except the e.s.d. in the dihedral angle between two l.s. planes) are estimated using the full covariance matrix. The cell e.s.d.'s are taken into account individually in the estimation of e.s.d.'s in distances, angles and torsion angles; correlations between e.s.d.'s in cell parameters are only used when they are defined by crystal symmetry. An approximate (isotropic) treatment of cell e.s.d.'s is used for estimating e.s.d.'s involving l.s. planes.
Refinement. Refinement of *F*^2^ against ALL reflections. The weighted *R*-factor *wR* and goodness of fit *S* are based on *F*^2^, conventional *R*-factors *R* are based on *F*, with *F* set to zero for negative *F*^2^. The threshold expression of *F*^2^ > σ(*F*^2^) is used only for calculating *R*-factors(gt) *etc*. and is not relevant to the choice of reflections for refinement. *R*-factors based on *F*^2^ are statistically about twice as large as those based on *F*, and *R*- factors based on ALL data will be even larger.

### Fractional atomic coordinates and isotropic or equivalent isotropic displacement parameters (Å^2^)

**Table:**

	*x*	*y*	*z*	*U*_iso_*/*U*_eq_	Occ. (<1)
F1	0.4769 (4)	−0.2372 (3)	0.2853 (4)	0.0698 (14)	0.710 (12)
F2	0.4529 (5)	−0.0522 (5)	0.2121 (3)	0.0731 (13)	0.710 (12)
F3	0.5816 (4)	−0.0554 (8)	0.3412 (5)	0.090 (2)	0.710 (12)
F1'	0.5251 (13)	−0.2132 (15)	0.3448 (14)	0.109 (5)	0.290 (12)
F2'	0.4491 (9)	−0.111 (2)	0.2122 (6)	0.111 (5)	0.290 (12)
F3'	0.5655 (11)	−0.0074 (18)	0.3219 (13)	0.092 (5)	0.290 (12)
O1	0.13692 (11)	0.11336 (15)	0.41856 (9)	0.0376 (4)	
O2	−0.17712 (11)	0.32576 (15)	0.48990 (9)	0.0348 (3)	
O3	0.02201 (12)	0.28785 (14)	0.67050 (9)	0.0367 (4)	
O4	−0.03869 (11)	0.50607 (14)	0.60863 (9)	0.0313 (3)	
N1	0.12414 (13)	0.41620 (18)	0.60530 (11)	0.0336 (4)	
H1	0.1242	0.4945	0.5755	0.040*	
C1	0.47480 (19)	−0.0960 (2)	0.29620 (17)	0.0477 (6)	
C2	0.38579 (17)	−0.0443 (2)	0.32947 (14)	0.0360 (5)	
C3	0.27526 (17)	−0.1023 (2)	0.29649 (13)	0.0360 (5)	
H3	0.2584	−0.1773	0.2543	0.043*	
C4	0.18957 (17)	−0.0526 (2)	0.32412 (13)	0.0332 (5)	
H4	0.1141	−0.0930	0.3009	0.040*	
C5	0.21432 (16)	0.0570 (2)	0.38617 (13)	0.0318 (4)	
C6	0.32588 (18)	0.1133 (2)	0.42067 (15)	0.0422 (5)	
H6	0.3437	0.1868	0.4640	0.051*	
C7	0.41034 (18)	0.0630 (2)	0.39219 (15)	0.0439 (5)	
H7	0.4862	0.1022	0.4158	0.053*	
C8	0.01688 (16)	0.0769 (2)	0.37353 (13)	0.0354 (5)	
H8A	0.0055	−0.0264	0.3792	0.043*	
H8B	−0.0081	0.1005	0.3096	0.043*	
C9	−0.05238 (17)	0.1595 (2)	0.41472 (13)	0.0329 (5)	
H9	−0.0182	0.2416	0.4485	0.039*	
C10	−0.15720 (18)	0.1256 (2)	0.40707 (14)	0.0381 (5)	
H10	−0.1877	0.0412	0.3745	0.046*	
C11	−0.23549 (17)	0.2024 (2)	0.44281 (13)	0.0337 (5)	
H11A	−0.3074	0.2308	0.3935	0.040*	
H11B	−0.2569	0.1392	0.4832	0.040*	
C12	−0.22437 (16)	0.3914 (2)	0.54436 (12)	0.0300 (4)	
C13	−0.33741 (17)	0.3764 (2)	0.53971 (13)	0.0354 (5)	
H13	−0.3893	0.3149	0.4967	0.042*	
C14	−0.37483 (18)	0.4512 (2)	0.59798 (14)	0.0394 (5)	
H14	−0.4523	0.4402	0.5948	0.047*	
C15	−0.30069 (18)	0.5415 (2)	0.66040 (14)	0.0392 (5)	
H15	−0.3271	0.5922	0.7001	0.047*	
C16	−0.18725 (17)	0.5581 (2)	0.66513 (13)	0.0347 (5)	
H16	−0.1360	0.6209	0.7076	0.042*	
C17	−0.14992 (16)	0.4832 (2)	0.60820 (12)	0.0296 (4)	
C18	0.03597 (16)	0.3915 (2)	0.63119 (12)	0.0290 (4)	
C19	0.22029 (18)	0.3175 (2)	0.62487 (16)	0.0446 (6)	
H19A	0.2818	0.3611	0.6091	0.067*	
H19B	0.1936	0.2300	0.5904	0.067*	
H19C	0.2504	0.2947	0.6884	0.067*	

### Atomic displacement parameters (Å^2^)

**Table:**

	*U*^11^	*U*^22^	*U*^33^	*U*^12^	*U*^13^	*U*^23^
F1	0.082 (2)	0.0421 (13)	0.117 (3)	0.0125 (13)	0.073 (2)	0.0034 (16)
F2	0.084 (3)	0.083 (2)	0.077 (2)	0.0261 (19)	0.059 (2)	0.019 (2)
F3	0.0276 (13)	0.136 (5)	0.100 (3)	−0.001 (2)	0.0170 (15)	−0.051 (3)
F1'	0.099 (7)	0.091 (7)	0.172 (12)	0.058 (6)	0.092 (8)	0.046 (7)
F2'	0.038 (4)	0.192 (13)	0.093 (7)	−0.003 (6)	0.011 (4)	−0.102 (8)
F3'	0.065 (7)	0.090 (7)	0.159 (11)	−0.039 (6)	0.087 (8)	−0.045 (7)
O1	0.0312 (7)	0.0435 (8)	0.0390 (8)	−0.0012 (6)	0.0141 (6)	−0.0093 (7)
O2	0.0333 (7)	0.0358 (8)	0.0412 (8)	−0.0074 (6)	0.0207 (6)	−0.0109 (6)
O3	0.0369 (8)	0.0314 (8)	0.0423 (8)	0.0002 (6)	0.0151 (6)	0.0082 (6)
O4	0.0279 (7)	0.0274 (7)	0.0399 (7)	0.0004 (6)	0.0139 (6)	0.0013 (6)
N1	0.0307 (8)	0.0351 (9)	0.0369 (9)	0.0017 (7)	0.0148 (7)	0.0039 (8)
C1	0.0362 (12)	0.0442 (13)	0.0672 (16)	0.0019 (11)	0.0243 (11)	0.0044 (13)
C2	0.0302 (10)	0.0354 (11)	0.0437 (11)	0.0024 (9)	0.0151 (9)	0.0051 (9)
C3	0.0347 (10)	0.0354 (11)	0.0385 (11)	−0.0001 (9)	0.0141 (9)	−0.0032 (9)
C4	0.0300 (10)	0.0346 (11)	0.0344 (10)	−0.0010 (8)	0.0111 (8)	−0.0010 (9)
C5	0.0302 (10)	0.0330 (10)	0.0327 (10)	0.0027 (8)	0.0121 (8)	0.0035 (8)
C6	0.0342 (11)	0.0405 (12)	0.0489 (12)	−0.0047 (10)	0.0118 (9)	−0.0112 (10)
C7	0.0285 (10)	0.0434 (12)	0.0573 (14)	−0.0043 (9)	0.0130 (10)	−0.0055 (11)
C8	0.0313 (10)	0.0384 (11)	0.0380 (11)	−0.0031 (9)	0.0145 (9)	−0.0050 (9)
C9	0.0347 (10)	0.0314 (10)	0.0335 (10)	−0.0015 (9)	0.0137 (8)	−0.0049 (8)
C10	0.0408 (11)	0.0331 (11)	0.0439 (12)	−0.0048 (9)	0.0200 (9)	−0.0095 (9)
C11	0.0334 (10)	0.0322 (10)	0.0367 (11)	−0.0048 (8)	0.0142 (8)	−0.0041 (8)
C12	0.0303 (10)	0.0313 (10)	0.0315 (10)	0.0019 (8)	0.0147 (8)	0.0007 (8)
C13	0.0297 (10)	0.0400 (12)	0.0382 (11)	−0.0026 (9)	0.0145 (9)	−0.0030 (9)
C14	0.0315 (10)	0.0457 (13)	0.0463 (12)	0.0046 (9)	0.0205 (9)	0.0035 (10)
C15	0.0408 (12)	0.0441 (12)	0.0378 (11)	0.0078 (10)	0.0206 (10)	0.0003 (10)
C16	0.0379 (11)	0.0333 (11)	0.0325 (10)	0.0025 (9)	0.0125 (9)	−0.0001 (9)
C17	0.0279 (9)	0.0291 (10)	0.0331 (10)	0.0013 (8)	0.0129 (8)	0.0029 (8)
C18	0.0271 (9)	0.0294 (10)	0.0284 (9)	−0.0008 (8)	0.0077 (8)	−0.0031 (8)
C19	0.0319 (11)	0.0475 (13)	0.0549 (13)	0.0068 (10)	0.0168 (10)	0.0000 (11)

### Geometric parameters (Å, º)

**Table:**

F1—C1	1.339 (3)	C6—H6	0.9500
F2—C1	1.351 (4)	C7—H7	0.9500
F3—C1	1.312 (5)	C8—C9	1.487 (3)
F1'—C1	1.363 (7)	C8—H8A	0.9900
F2'—C1	1.287 (8)	C8—H8B	0.9900
F3'—C1	1.337 (9)	C9—C10	1.300 (3)
O1—C5	1.361 (2)	C9—H9	0.9500
O1—C8	1.438 (2)	C10—C11	1.488 (3)
O2—C12	1.373 (2)	C10—H10	0.9500
O2—C11	1.429 (2)	C11—H11A	0.9900
O3—C18	1.210 (2)	C11—H11B	0.9900
O4—C18	1.379 (2)	C12—C13	1.384 (3)
O4—C17	1.395 (2)	C12—C17	1.401 (3)
N1—C18	1.329 (2)	C13—C14	1.387 (3)
N1—C19	1.451 (3)	C13—H13	0.9500
N1—H1	0.8800	C14—C15	1.379 (3)
C1—C2	1.479 (3)	C14—H14	0.9500
C2—C7	1.383 (3)	C15—C16	1.390 (3)
C2—C3	1.387 (3)	C15—H15	0.9500
C3—C4	1.379 (3)	C16—C17	1.369 (3)
C3—H3	0.9500	C16—H16	0.9500
C4—C5	1.392 (3)	C19—H19A	0.9800
C4—H4	0.9500	C19—H19B	0.9800
C5—C6	1.393 (3)	C19—H19C	0.9800
C6—C7	1.376 (3)		
			
C5—O1—C8	117.30 (15)	H8A—C8—H8B	108.4
C12—O2—C11	117.06 (15)	C10—C9—C8	123.63 (19)
C18—O4—C17	116.65 (14)	C10—C9—H9	118.2
C18—N1—C19	121.73 (18)	C8—C9—H9	118.2
C18—N1—H1	119.1	C9—C10—C11	128.22 (19)
C19—N1—H1	119.1	C9—C10—H10	115.9
F2'—C1—F3'	104.5 (10)	C11—C10—H10	115.9
F3—C1—F1	107.3 (4)	O2—C11—C10	108.98 (16)
F3—C1—F2	104.9 (4)	O2—C11—H11A	109.9
F1—C1—F2	100.1 (3)	C10—C11—H11A	109.9
F2'—C1—F1'	113.7 (8)	O2—C11—H11B	109.9
F3'—C1—F1'	99.7 (8)	C10—C11—H11B	109.9
F2'—C1—C2	119.7 (6)	H11A—C11—H11B	108.3
F3—C1—C2	116.1 (4)	O2—C12—C13	125.72 (17)
F3'—C1—C2	109.8 (7)	O2—C12—C17	115.42 (16)
F1—C1—C2	115.2 (2)	C13—C12—C17	118.84 (18)
F2—C1—C2	111.6 (3)	C12—C13—C14	119.89 (19)
F1'—C1—C2	107.4 (5)	C12—C13—H13	120.1
C7—C2—C3	119.2 (2)	C14—C13—H13	120.1
C7—C2—C1	121.03 (19)	C15—C14—C13	120.68 (19)
C3—C2—C1	119.8 (2)	C15—C14—H14	119.7
C4—C3—C2	120.9 (2)	C13—C14—H14	119.7
C4—C3—H3	119.5	C14—C15—C16	119.8 (2)
C2—C3—H3	119.5	C14—C15—H15	120.1
C3—C4—C5	119.58 (19)	C16—C15—H15	120.1
C3—C4—H4	120.2	C17—C16—C15	119.53 (19)
C5—C4—H4	120.2	C17—C16—H16	120.2
O1—C5—C4	124.58 (18)	C15—C16—H16	120.2
O1—C5—C6	115.86 (18)	C16—C17—O4	119.88 (17)
C4—C5—C6	119.54 (19)	C16—C17—C12	121.22 (18)
C7—C6—C5	120.2 (2)	O4—C17—C12	118.71 (17)
C7—C6—H6	119.9	O3—C18—N1	126.96 (18)
C5—C6—H6	119.9	O3—C18—O4	123.69 (17)
C6—C7—C2	120.6 (2)	N1—C18—O4	109.33 (16)
C6—C7—H7	119.7	N1—C19—H19A	109.5
C2—C7—H7	119.7	N1—C19—H19B	109.5
O1—C8—C9	108.10 (16)	H19A—C19—H19B	109.5
O1—C8—H8A	110.1	N1—C19—H19C	109.5
C9—C8—H8A	110.1	H19A—C19—H19C	109.5
O1—C8—H8B	110.1	H19B—C19—H19C	109.5
C9—C8—H8B	110.1		
			
F2'—C1—C2—C7	−132.8 (10)	C5—O1—C8—C9	−175.09 (16)
F3—C1—C2—C7	12.8 (5)	O1—C8—C9—C10	−160.9 (2)
F3'—C1—C2—C7	−11.9 (10)	C8—C9—C10—C11	−177.9 (2)
F1—C1—C2—C7	139.4 (4)	C12—O2—C11—C10	−165.39 (16)
F2—C1—C2—C7	−107.3 (3)	C9—C10—C11—O2	−0.2 (3)
F1'—C1—C2—C7	95.6 (11)	C11—O2—C12—C13	−19.5 (3)
F2'—C1—C2—C3	46.2 (10)	C11—O2—C12—C17	161.91 (17)
F3—C1—C2—C3	−168.2 (4)	O2—C12—C13—C14	−178.79 (18)
F3'—C1—C2—C3	167.0 (10)	C17—C12—C13—C14	−0.2 (3)
F1—C1—C2—C3	−41.6 (4)	C12—C13—C14—C15	0.4 (3)
F2—C1—C2—C3	71.7 (3)	C13—C14—C15—C16	0.1 (3)
F1'—C1—C2—C3	−85.4 (11)	C14—C15—C16—C17	−0.7 (3)
C7—C2—C3—C4	1.2 (3)	C15—C16—C17—O4	175.88 (17)
C1—C2—C3—C4	−177.75 (19)	C15—C16—C17—C12	0.8 (3)
C2—C3—C4—C5	−0.2 (3)	C18—O4—C17—C16	117.69 (19)
C8—O1—C5—C4	−12.6 (3)	C18—O4—C17—C12	−67.1 (2)
C8—O1—C5—C6	169.01 (18)	O2—C12—C17—C16	178.33 (17)
C3—C4—C5—O1	−179.48 (18)	C13—C12—C17—C16	−0.4 (3)
C3—C4—C5—C6	−1.1 (3)	O2—C12—C17—O4	3.2 (3)
O1—C5—C6—C7	179.85 (19)	C13—C12—C17—O4	−175.48 (17)
C4—C5—C6—C7	1.3 (3)	C19—N1—C18—O3	−1.9 (3)
C5—C6—C7—C2	−0.3 (3)	C19—N1—C18—O4	176.30 (17)
C3—C2—C7—C6	−1.0 (3)	C17—O4—C18—O3	−18.9 (3)
C1—C2—C7—C6	178.0 (2)	C17—O4—C18—N1	162.82 (15)

### Hydrogen-bond geometry (Å, º)

**Table:**

*D*—H···*A*	*D*—H	H···*A*	*D*···*A*	*D*—H···*A*
N1—H1···O2^i^	0.88	2.22	3.072 (2)	163

Symmetry code: (i) −*x*, −*y*+1, −*z*+1.
